# Books and Pamphlets Received

**Published:** 1885-09-20

**Authors:** 


### BOOKS AND PAMPHLETS RECEIVED.

*A Treatise on Epidemic Cholera and allied diseases.* By A. B. Palmer, M. D., LL. D., Professor of Practice of Medicine and Clinical Medicine in the College of Medicine and Surgery in the University of Michigan ; author of a work on the Science and Practice of Midicine, etc. Ann Arbor, Mich.: Register Publishing House. 1885. 224 pages. Cloth.

The object of the work, the author states, " has been to collect and present in an acceptable form, an account of the more important facts observed, the investigations made, and the more authoritative opinions expressed respecting the disease from the earliest authenticated history up to the present time." The author has himself passed through three cholera seasons in Chicago, and speaks experimentally on some points. The work is interesting, instructive, and very timely in view of the prevalence of the disease in Europe, and its probable visitation to this country at an early period.

*Inebriism.* A pathological and psychological study by T. L. Wright, M. D., member of the American Association for the Cure of Inebriates. Columbus, Ohio: William G. Hubbard. 1885.

The above is an ably written work of 222 pages, neatly printed. It contains thoughts of grave importance and deep interest touching the causes and pathological conditions of alcoholic inebriety. In view of the wide spread and growing sentiment now prevailing in favor of prohibitory laws, the work is timely and appropriate, and we trust will be warmly welcomed by the friends of temperance, and by medical men especially, as furnishing important principles for their guidance in the use of alcoholic stimulants as remedies, and as furnishing material with which to combat the arguments of those who oppose temperance reform.

*A Practical Treatise on Nasal Catarrh* and allied diseases. By Beverly Robinson, A. M., M. D., (Paris), Clinical Professor of Medicine at the Bellevue Hospital Medical College, New York ; Physician to Saint Luke's and Charity Hospitals, etc. Second edition. Revised and enlarged. With 152 illustrations. New York: William Wood & Co. 1885.

Five chapters have been added in this volume to this excellent work of Professor Robinson. These refer to aural complications, to deflections, etc., of the nasal system, to ulcerous coryza, vegetations, etc., of the pharynx, and to nasal polypi. It is an eminently practical and useful work, and will prove very acceptable both to the specialist and to the general practitioner, as a book of reference in this perplexing department of Medical Science.

*Micro-Chemistry of Poisons,* including their Physocological, Pathological, and Legal Relations ; with an Appendix of the Detection and Microscopic Discrimination of Blood ; adapted to the use of the Medical Jurist, Physician and general Chemist. By Theodore G. Wormley, M.D., Ph. D., and LL.D., Professor of Chemistry and Toxicology in the Medical Department of the University of Pennsylvania. With ninety-six illustrations on steel. Second edition. 740 octavo pages. Philadelphia: J. B. Lippincott Company. 1885.

" This work," says the author, " has been thoroughly revised and much enlarged in matter, especially by the addition of illustrated cases, largely American, and by new tests and methods of recovery of poisons from organic mixtures, and also by the addition of an entirely new chapter on Gelsemium Poisoning, and an appendix on the nature, detection, and microscopic discrimination of Blood. The other subjects mentioned are poisoning by potassum, chlorate, arsenic in medicine and in fabrics ; nature of Ptomaines, etc."

The illustrations in the latter part of the work are beautiful and interesting. The chemical nomenclature has been revised, and now conforms to the more recent views of Chemists, and we are glad to note that the metric system is not used. We commend the work as eminently able, practical, and useful.

*Eaythroxylon Coca.* To the Medical Profession. Philadelphia, Pa.: William P. Warner & Co.

A very interesting monograph, containing the latest and most important information in reference to coca, with a beautiful illustration of the plant.

*Modern Therapeutics of the Diseases of Children*, with Observations on the Hygiene of Infancy. By Joseph E. Edwards, M. D., Editor of the Annals of Hygiene; Associate Editor of the Medical and Surgical Reporter; author of Bright's Disease and i s Treatment; Fellow of the College of Physicians and Surgeons, etc. Philadelphia: D. G. Brinton, 115 South Seventh street. 1885.

The work contains 346 octavo pages. The author states that the purpose of the work is, to present in a concise manner the results of the modern application of Therapeutics to the Diseases of Children. It is one of a series of works in which the author is endeavoring to give the details of the most eminent specialists in this branch. We find it replete with numerous excellent formulæ and valuable practical suggestions, drawn, in large measure, frum eminent sources. We recommend it as a highly valuable addition to our works in the important department of Diseases of Children.

*N. W. Ayer & Son's American Newspaper Annual*, containing a catalogue of American Newspapers; a carefully prepared list of all Newspapers and Periodicals published in the United States, Territories, and the Dominion of Canda, with valuable information regarding their circulation, issue, date of establishment, political or other distinctive features, and advertising rates; together with the population of the cities and towns, as well as of the counties in which they are pubiished; a list of all Newspapers in the United States and Canada which insert advertisements, arranged by counties, with a description of each State, Territory, and County in the United States; giving the location, area, character of surface and soil, chief products, and manufactures; also separate lists of all Religious and Agricultural publications, the various class publications, and all Newspapers published in Foreign languages, omitting those which do not insert advertisements. Philadelphia, Pa.: N. W. Ayer & Son, Newspaper Advertising Agents, Times Building, Chestnut and Eighth strcets. 1885.

This is a very large and elaborate work of nearly 1,000 pages, which must have required immense care and labor in its preparation. The above enumeration of its contents will suffice to show the immense scope and usefulness of the work. Upon examining it, we find that it is as accurate as can well be expected, as upon some points information is obtained with great difficulty, and often, from necessity, only an approximate statement can be given. This applies especially to the circulation of journals and newspapers, in which the honest publisher often refuses to report his list because he knows that many others competing with him will not report honestly. Upon the whole, however, we commend the above work as one of great importance and usefulness.

